# Antimicrobial Stewardship Intervention for the Family Caregiver Attending Primary Health Care Setting: A Quasi-Experimental Study

**DOI:** 10.3390/antibiotics13121145

**Published:** 2024-11-29

**Authors:** Ammena Y. Binsaleh, Mahmoud S. Abdallah, Basma Mohamed Osman, Mostafa M. Bahaa, Nawal Alsubaie, Thanaa A. Elmasry, Mohamed Yasser, Mamdouh Eldesoqui, Abdel-Naser Abdel-Atty Gadallah, Manal A. Hamouda, Nashwa Eltantawy, Fatma A. Mokhtar, Ramy M. El Sabaa

**Affiliations:** 1 Department of Pharmacy Practice, College of Pharmacy, Princess Nourah bint Abdulrahman University, P.O. Box 84428, Riyadh 11671, Saudi Arabia; 2 Department of Clinical Pharmacy, Faculty of Pharmacy, University of Sadat City (USC), Sadat City 32879, Egypt; 3 Department of PharmD, Faculty of Pharmacy, Jadara University, Irbid 21110, Jordan; 4 Community Health Nursing, Faculty of Nursing, Cairo University, Cairo 11562, Egypt; 5 Department of Community Health Nursing, Faculty of Nursing, The British University in Egypt, El Sherook City 11837, Egypt; 6 Pharmacy Practice Department, Faculty of Pharmacy, Horus University, New Damietta 34517, Egypt; 7 Pharmacology and Toxicology Department, Faculty of Pharmacy, Tanta University, Tanta 31527, Egypt; 8 Department of Pharmaceutics, Faculty of Pharmacy, Port Said University, Port Said 42526, Egypt; 9 Department of Pharmaceutics and Industrial Pharmacy, Faculty of Pharmacy, Horus University, New Damietta 34518, Egypt; 10 Department of Pharmaceutics, Faculty of Pharmacy, East Port Said National University, Port Said 42526, Egypt; 11 Department of Basic Medical Sciences, College of Medicine, AlMaarefa University, Riyadh 13713, Saudi Arabia; 12 Internal Medicine Department, Faculty of Medicine, Menofia University, Shebin El-Kom 32511, Egypt; 13 Department of Clinical Pharmacy, Faculty of Pharmacy, Menofia University, Shebin El-Kom 32511, Egypt; 14 Department of Pharmacy Practice, Faculty of Pharmacy and Drug Technology, Egyptian Chinese University, Cairo 11786, Egypt; 15 Department of Pharmacognosy, Faculty of Pharmacy, El Saleheya El Gadida University, El Saleheya El Gadida 44813, Egypt; 16 Fujairah Research Centre, Sakamkam Road, Sakamkam, Fujairah 00000, United Arab Emirates

**Keywords:** AMR, family caregiver, knowledge, self-reported practice, antimicrobial stewardship

## Abstract

**Background:** Antimicrobial resistance (AMR) has been identified as one of the top ten public health threats facing humanity. **Aim:** The purpose of this study was to assess the effect of an antimicrobial stewardship educational intervention on family caregivers’ knowledge and practices in primary healthcare settings in Egypt. **Methods:** A quasi-experimental, one-group pretest-posttest design involving a sample of 300 family caregivers attending family health centers. The data were collected using questionnaires that assessed caregiver knowledge and self-reported practices regarding AMR before and after the intervention (primary outcomes). The intervention combined tailored knowledge and practice components that carefully evaluated participants’ knowledge regarding AMR, health risk, antibiotic usage, and prevention of infection. Furthermore, their practice of using antibiotics, including previous antibiotic exposure, their antibiotic use during the past year, reasons for taking antibiotics, ways of obtaining antibiotics, and reasons for discontinuing of antibiotic therapy were also recorded. **Results:** The pre-intervention assessment revealed poor knowledge and practice regarding antibiotic use. Post-intervention, mean knowledge scores increased significantly from 18.36 to 23.28 (t = 19.5, *p* < 0.0001), while mean practice scores improved from 9.83 to 12.37 (t = 6.4, *p* < 0.0001). **Conclusions:** The intervention successfully improved caregivers’ knowledge and practices regarding AMR. However, there are some limitations that could affect the generalization, and the impact of the results such as the relatively small sample size recruited from a single center, lack of a control group, reliance on self-reported data, and lack of long-term follow-up. Future studies should aim to address these constraints in order to assess the intervention’s effectiveness.

## 1. Introduction

Antimicrobial resistance (AMR) is a growing public health concern. The emergence of AMR is a rapidly developing global threat that greatly affects our ability to deliver effective healthcare and results in a financial burden [1,2,3]. The global demand for antimicrobials is increasing, and the inadequate use leads to the abuse of vast amounts of antimicrobials [4].

Antimicrobial stewardship (AMS) aims to preserve antibiotic effectiveness through interventions that are persuasive (education and feedback), restrictive (guidelines and controls), and structural (governance and audits). The successful implementation of AMS is expected to limit the negative impacts of antibiotic use, such as drug toxicity and AMR [1].

AMR in community settings is driven by a variety of causes, including antimicrobial abuse, a lack of clean water, inadequate sanitation, restricted access to quality medical supplies, and a lack of awareness [5]. Previous evidence showed that the overall levels of knowledge and understanding of AMR amongst the public are generally low, and the general population often lacks an understanding of their possible contribution to the development of AMR [2].

Public health steward educational interventions aimed at raising awareness and changing people’s behavior in this area are very important because people and animals overuse and misuse antibiotics (for example, not following doctors’ orders, not taking or demanding antimicrobial prescriptions for colds and flu, and not properly disposing of leftover antimicrobials) are some of the things that cause AMR [6,7].

Educating family caregivers is a viable and easily implementable real-world solution against antibiotic resistance [8,9,10]. Implementing AMS interventions in primary healthcare settings with a focus on educating family caregivers is essential for effectively addressing the AMR threat [11,12]. Family caregivers are key stakeholders in healthcare decision-making. AMS programs can promote responsible antibiotic use, curb antimicrobial resistance, and influence household antibiotic use. Educating these caregivers is a practical solution against antibiotic resistance, impacting future generations [8,9,10].

Healthcare professionals (HCWs) should be considered reliable sources of information since they counsel patients on the use of antibiotics, making them the ideal people to investigate knowledge, attitude, and practice (KAP) on AMR. Consequently, in 2020, a Riyadh-based research evaluating physicians’ knowledge of the Antimicrobial Stewardship Program (ASP) revealed that approximately 56% of them could name the program and 65% were aware of the distinction between bacteriostatic and bactericidal drugs [13]. Additionally, over 50% of the HCWs were observed to be unaware of ASP [14]. A statewide study carried out in the Kingdom of Saudi Arabia found that the setting up of ASPs was inefficient and that the following variables contributed to this ineffectiveness: lack of knowledge, ASP teams, experience with infectious diseases, and technical resources [15]. It was discovered that there was another study that evaluated nursing students’ knowledge of AR and antibiotic utilization during COVID-19 [16]. According to this study, nursing students at the International Islamic University Malaysia have good KAP when it comes to antibiotic use and antimicrobial resistance. Additionally, earlier studies investigated healthcare workers’ knowledge, attitudes, and actions about antibiotic usage and resistance in Italy and 30 other European countries in 2019 [17,18]. They concluded that KAP on antibiotic use and resistance varied among HCW. These results highlighted the necessity of training and educational initiatives for all professional groups.

Educational interventions appear to be an integral component of other steward intervention. There is a need for AMR awareness interventions directed at the public, as well as the development of a uniform, globally consistent set of AMR messages that could be tailored to meet the specific demands of local settings. Public awareness programs should incorporate antibiotic-related information, particularly targeting the population from a lower socioeconomic class [2].

Therefore, the main objective of this study is to combat AMR by implementing a targeted educational intervention for family caregivers in primary healthcare settings. By addressing this crucial demographic, the research fills a critical gap in current AMR prevention strategies. Furthermore, engaging caregivers in AMR prevention efforts creates a ripple effect, potentially influencing broader community attitudes and practices. By fostering a culture of responsible antimicrobial use among family caregivers, the research lays the groundwork for sustainable AMR mitigation strategies, potentially informing policy and practice in primary healthcare settings globally. Ultimately, this research has far-reaching implications, extending beyond immediate health outcomes to safeguard the effectiveness of antibiotics for future generations. In line with these objectives, this study aimed to evaluate the effect of antimicrobial stewardship intervention on family care giver knowledge and practice regarding antibiotic misuse.

## 2. Results

### 2.1. Demographic and Social Data of Family Caregiver

Table 1 summarizes the demographic profile of family caregivers: 98% were female, 26% were aged 25–35 years, 44% had middle education, 29.7% had higher education, and 4% were illiterate. Most participants (80%) were married. Employment status showed that 47% were employed and 39.7% were housewives. Income distribution revealed 41% not enough, while 15% reported enough and savings.

### 2.2. Distribution of Family Caregivers Related to Self-Reported Commonly Used Antibiotics

**Figure 1:**
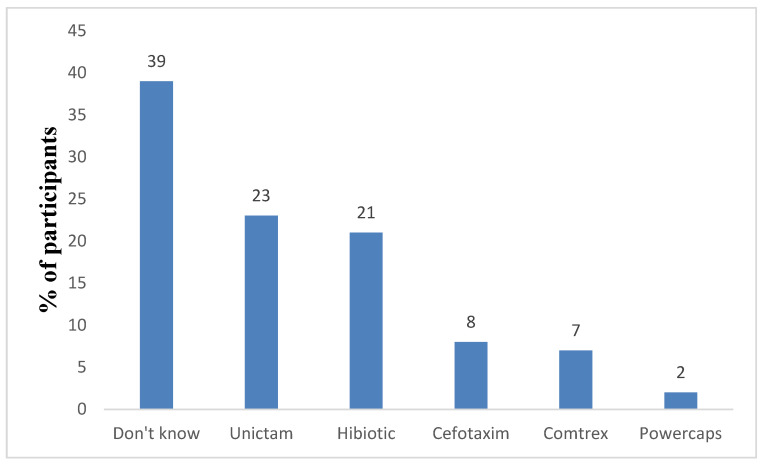
Distribution of family caregivers related to self-reported commonly used antibiotics.

Figure 1 shows that 39% of family caregivers were unable to recall their commonly used antibiotics. Among those who could name specific antibiotics, 23% reported Unictam (Ampicillin/Sulbactam combination) as commonly used, 21% reported Hibiotic, and 8% reported Cefotaxime, while 2% incorrectly referenced Power Caps and 7% misidentified Comtrex as an antibiotic. After the intervention, only 10% of the family caregivers were unable to recall the used antibiotics, and no one incorrectly mentioned their used antibiotics. Furthermore, nobody reported using Cefotaxime.

### 2.3. Distribution of the Family Care Giver Related to Their Knowledge in the Pre and Post-Test

There was a significant improvement in family caregivers’ antibiotic knowledge post-intervention. Correct identification of antibiotics treating viral infections rose from 43.0% to 97.3%. Understanding antibiotic resistance increased from 67.7% to 97.7%. Most statements showed significant improvements, except for “excessive intake of antibiotics can reduce the child’s immunity”, which increased from 87.7% to 97.3% but was not statistically significant (*p* = 0.22) (Table 2).

**Figure 2:**
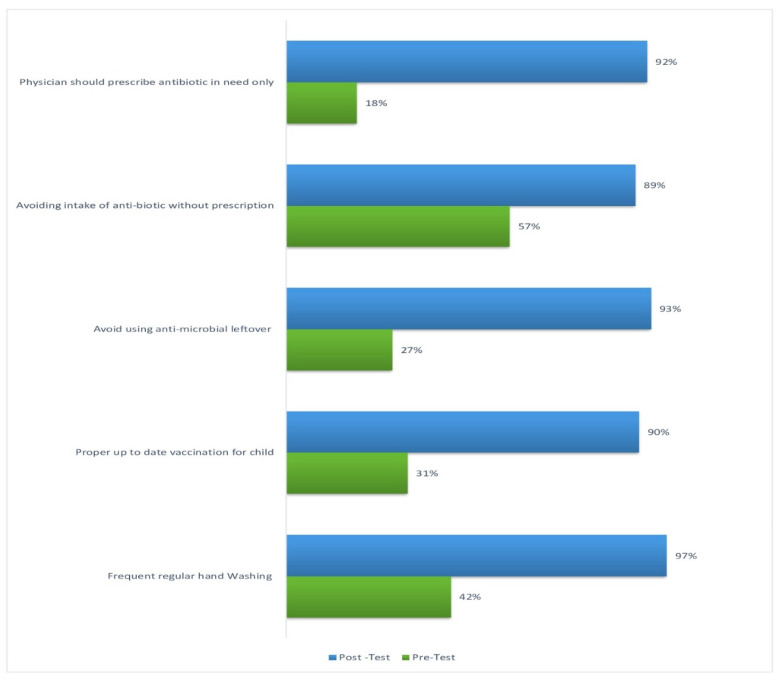
Distribution of the family caregiver regarding their knowledge on antimicrobial resistance mitigation in the pre and post-test (*n* = 300).

Figure 2 illustrates significant improvements in knowledge about mitigating antimicrobial resistance post-intervention. Regular hand washing increased from 42% to 97%, while proper child vaccination rose from 31% to 90%. Avoiding leftover antimicrobials and unprescribed antibiotics increased from 27% to 93% and 57% to 89%, respectively. The belief that physicians should prescribe antibiotics only, when necessary, rose from 18% to 92%.

### 2.4. Effect of Intervention on Antimicrobial Usage

**Figure 3:**
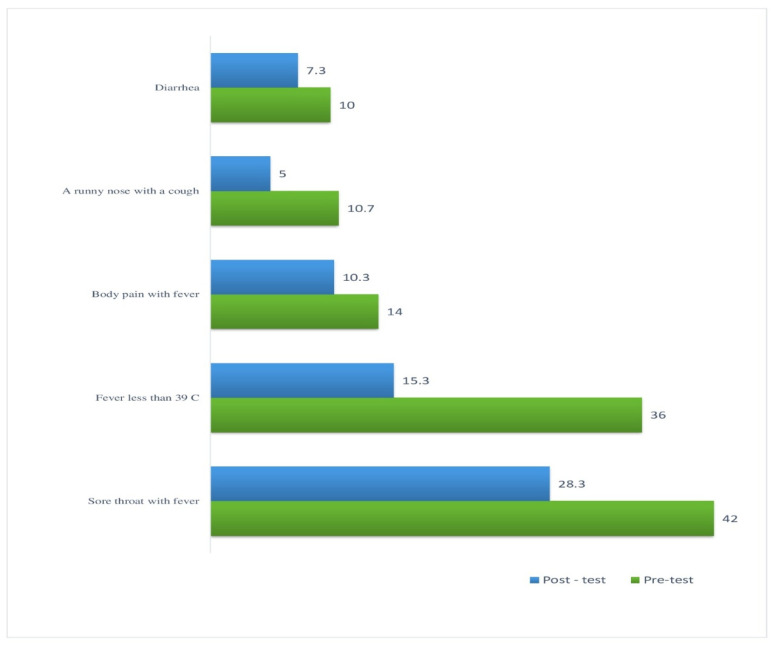
Distribution of family care giver related to common symptoms indicating self-prescriptions of antibiotics (*n* = 300).

Figure 3 confirmed that antimicrobial self-prescription shifted, with reductions observed for sore throat with fever (from 42% to 28.3%), fever less than 39 °C (from 36% to 15.3%), runny nose with cough (from 10.5% to 5%), and diarrhea (from 10% to 7.3).

### 2.5. Distribution of the Family Care Giver Related to Their Practices in the Pre and Post-Test

There were significant changes in family caregivers’ antibiotic use practices post-intervention. The percentage of caregivers considering antibiotic prices when making choices nearly doubled from 36.0% to 69.7%. Conversely, those who considered antibiotic types decreased from 35.3% to 17.7%. Furthermore, caregivers’ intentional changes in antibiotic type or dose decreased from 36.7% to 25.7%. The data reveals significant changes in caregivers’ antibiotic-related practices post-intervention (Table 3). Regarding dose calculation, consultation with doctors increased from 40.0% to 65.7%, while reliance on pharmacists decreased from 24.0% to 14.7%.

Regarding post-intervention antibiotics practice by family caregivers for their children, the percentage of caregivers who never gave antibiotics without a prescription increased from 30.0% to 51.7%, while those who did so “rarely” or “usually” decreased. Pediatricians’ phone prescriptions also declined, with “usually” dropping from 32.0% to 19.3%. Caregivers became less likely to share their own antibiotics with children, increasing from 73.0% to 76.3%. Notably, adherence to completing the full course of antibiotics improved, rising from 33.7% to 48.0%.

**Figure 4:**
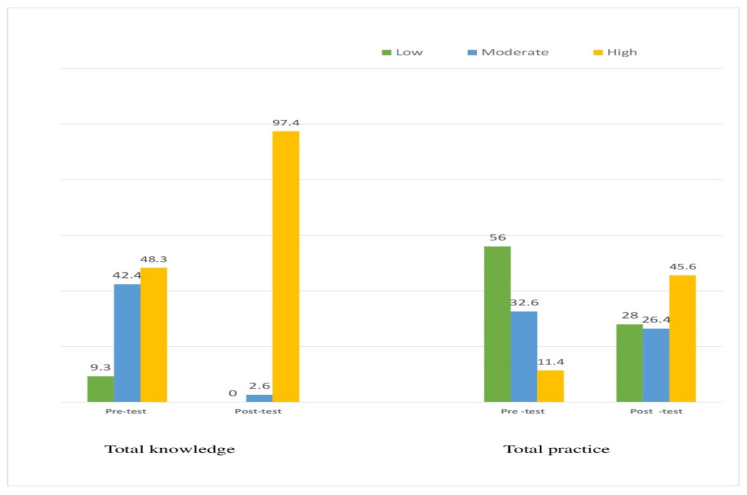
Distribution of the family care giver related to their total knowledge and practice scores in the pre and post-test (*n* = 300).

Figure 4 demonstrates a marked improvement in family caregivers’ total knowledge scores post-intervention. Pre-test results showed 9.3% with poor knowledge, 42.4% with fair knowledge, and 48.3% with high knowledge. Post-intervention, poor knowledge decreased to 0%, fair knowledge decreased to 2.6%, and high knowledge increased significantly to 97.4%. Also, there is a marked improvement in family caregivers’ antibiotic practices post-intervention. Pre-test results showed 56% with low practice scores, 32.6% with moderate scores, and 11.4% with high scores. Post-intervention, low practice scores decreased to 28%, moderate scores decreased to 26.4%, and high practice scores increased significantly to 45.6%.

### 2.6. Mean Scores of Total Knowledge and Practice Scores of Family Caregiver in Pre and Post-Test

Table 4 shows significant improvements in family caregivers’ total knowledge and practice scores post-intervention. The mean knowledge score increased from 18.36 to 23.28 (t = 19.5, *p* = 0.0001), while the mean practice score rose from 9.83 to 12.37 (t = 6.4, *p* = 0.0001). Both improvements are statistically significant (*p* < 0.05), with the higher t-value for knowledge scores suggesting a more pronounced effect on knowledge acquisition. Subgroup analysis revealed that knowledge and practice scores increased significantly in all categories of education (*p* < 0.05), as shown in Table 4.

Antimicrobial resistance (AMR) poses a significant global public health threat, largely fueled by inappropriate antibiotic use [19]. Family caregivers often harbor misconceptions about antibiotics, such as their efficacy against viral infections or the acceptability of incomplete treatment courses [20]. These misunderstandings can result in unwarranted antibiotic demands and poor adherence to prescribed regimens, exacerbating the AMR crisis.

Our findings revealed poor pre-intervention knowledge and self-reported practices regarding antibiotic use among family caregivers, highlighted the urgent need for healthcare professionals to take a more active role in patient education, focusing on proper antibiotic administration and usage. This is consistent with findings from Hassan et al., (2023), who conducted a comprehensive study across 11 Middle Eastern and North African countries, demonstrating limited knowledge towards antibiotic use and resistance among Arab populations [21]. Similarly, an Egyptian study by Hafez et al., (2024), who evaluated the effectiveness of a nursing-based intervention aimed at optimizing antibiotic use among mothers of children under 5 years, found that 64% of mothers had an unsatisfactory level of knowledge [19].

The fact that 9% of participants were unclear about antibiotics and 39% of participants couldn’t name the antibiotic indicates a fundamental problem with participants’ antibiotic literacy. The improvement in these data following the intervention suggested that it was helpful in addressing basic concepts like the definition of an antibiotic and its proper usage. Furthermore, the inappropriate use of a Watch antibiotic like cefotaxime is concerning because these antibiotics are intended for specific, careful use to avoid resistance, and their misuse can contribute to larger public health problems. Family caregivers’ awareness of cefotaxime use rose after the intervention. Our findings were consistent with earlier reports [22,23]. According to Abu-Ajaleh et al., increasing doctors’ and pharmacists’ education and awareness improved the use of Watch antibiotics [22]. Furthermore, Apisarnthanarak et al. reported that education and an antibiotic-control program were an effective and cost-efficient technique for optimizing antibiotic use at a tertiary care hospital in Thailand. The usage of unsuitable antibiotics, especially third generation cephalosporins, was dramatically reduced [24]. Thus, our study’s findings demonstrated the value of educational initiatives in lowering the use of Watch antibiotics.

The results of this study revealed that the total knowledge scores regarding antimicrobial stewardship had significantly improved following the educational intervention. Also, subgroup analysis revealed that knowledge and practice scores increased significantly in all categories of education compared to pre-test. This significant shift in knowledge levels underscores the potential of targeted stewardship educational programs to promote more responsible antibiotic use among family caregivers. Similarly, Hafez et al. (2024) showed significant improvements in the participants’ knowledge levels both pre- and post-intervention, demonstrating a satisfactory increase from 36% to 78% after the intervention, with a *p*-value of less than 0.05 [19]. Paradoxically, the “can’t read and write” category had a higher sub-score knowledge and practices compared to the “higher education” category. These findings could be the result of the possibility of changing beliefs, thoughts, and perceptions of illiterate people compared to higher education ones. Also, the ease of convincing and the complete commitment of the “can’t read and write” category to follow instructions may be responsible for this higher score. Previous studies reported that people with higher education had higher knowledge scores compared to illiterate and low-level education [13,25]. So, further studies will be required to address this issue.

Aika & Enato (2023) conducted a comparative study on the effectiveness of one-on-one and group educational interventions in improving pediatric caregivers’ antibiotic knowledge and use at pediatric outpatient clinics in Nigerian Pidgin [9]. They found significant improvements in both knowledge and practice scores. Knowledge scores increased from 36.1 ± 6.467 to 46.7 ± 4.027 (*p* ≤ 0.0001). Similarly, a pilot study by Appiah et al. (2022) evaluated the effectiveness of antimicrobial resistance awareness interventions for schoolchildren and parents, finding that parents who participated in the animation event and their children’s school-based storytelling intervention improved their knowledge of antibiotic efficacy, with correct responses increasing from 50% to 88% at the endline (χ^2^ = 0.711, *p* = 0.021) [26].

Our results revealed that family caregivers demonstrated increased awareness of antibiotic resistance following the intervention, highlighting this substantial improvement in understanding antibiotic resistance and promoting judicious antibiotic use. This finding aligns with a study by Ramanarayanan et al. (2024), which reported that educational programs effectively increased awareness of antibiotic resistance among university students [27].

The lack of statistically significant changes in understanding of antibiotics’ effects on children’s immunity could be attributed to preconceived assumptions or preconceptions of the participants about antibiotics and immunity that were not fully addressed in the intervention, making it difficult to change their perceptions [28]. As a result, experimenting with a range of strategies, such as supplementing instructional information with relevant examples and using visual aids to illustrate complex topics, may yield better outcomes in these specific knowledge areas [29].

The study’s findings showed that caregivers had a better awareness that antibiotics are ineffective against viral infections, which helped to refute a prevalent myth that contributes to antibiotic abuse. The findings were consistent with reports from Aika & Enato (2023), which showed that educational programs dramatically lowered parents’ expectations for antibiotics in viral infections [9].

The total practice scores regarding antimicrobial stewardship had significantly improved after the educational intervention in this study. This is consistent with findings from Shen et al. (2021), who assessed the effectiveness of an intervention for residents in rural China and reported greater improvements in response to self-reported practices regarding antibiotic use in humans within the intervention group, as well as in practice related to antibiotic use in pigs [6].

While the immediate post-intervention results are promising, it is important to consider the long-term retention of these improved practices, which is a notable shortcoming of this study. A study by Afzal et al. (2023) in China demonstrated that the positive effects of an educational intervention on antibiotic use practices among caregivers were sustained even after six months, suggesting the potential for a lasting impact [30]. This trend is supported by a systematic review by Satterfield et al. (2020), which concluded that educational interventions are valuable components of antimicrobial stewardship programs, especially when tailored to specific audiences like family caregivers [23].

Reduction in self-prescription antibiotic use is a substantial improvement with observed decrease for sore throat with fever, runny nose with cough, sore throat with fever, fever less than 39 °C and runny nose with cough, and diarrhea. This aligns with findings from a systematic review by Fuller et al. (2023), which showed that educational interventions can effectively reduce non-prescription antibiotic use in low- and middle-income countries [8]. Similarly, a study by Lam et al. (2021) found that community-based interventions can significantly decrease non-prescription antibiotic use in low- and middle-income countries [3]. These trends generally suggest improved antibiotic stewardship practices and public awareness. However, the persistence of some self-prescription, particularly for sore throat with fever, indicates ongoing need for education and interventions to further reduce unnecessary antibiotic use.

The decrease in pediatricians’ phone prescriptions reported in this study align with the findings from Wittman et al. (2024) which indicated that antibiotic prescribing rates during pediatric telemedicine visits were higher compared to in-person consultations. This underscores the importance of reducing unnecessary prescriptions in remote consultations [31].

After the intervention, some answers suggest that family caregivers understand the importance of adhering to full antibiotic courses. This aligns with findings from Almomani et al. (2023), who demonstrated that pharmacist-led educational interventions can significantly enhance adherence to prescribed short-term antibiotics among adult patients diagnosed with acute infection and prescribed a short-term antibiotic course at a tertiary referral hospital in Jordan [32]. Additionally, Biniek et al. (2024) emphasized the importance of adherence to antibiotic prescription guidelines in community pharmacies [33]. Current study results convey a reduced sharing of their own antibiotics with children after educational intervention. Similarly, a study by Barber et al. (2017) highlighted the dangers of antibiotic sharing and emphasized the importance of education in reducing this practice [34].

The overall impact of educational interventions collectively demonstrates the effectiveness of educational interventions in improving antibiotic use knowledge and practices. This is supported by Aika & Enato (2023) [9]. Educational programs and public messaging campaigns have resulted in substantial declines in antibiotic use and should be used as models for succeeding efforts to curb inappropriate antibiotic use and subsequent antibiotic resistance [35].

#### Strengths and Limitations

The study’s strengths include its focus on a crucial group for antibiotic stewardship, comprehensive assessment of multiple aspects of antibiotic use, with practical implications for public health. However, the claimed gains in self-reported practices must be contextualized inside real-world behaviors to determine their genuine impact. While self-reports can show a favorable shift in attitudes and intentions, they do not always correspond to real behavioral changes over time. Furthermore, there are some limitations that could affect the generalization, and the impact of the results include the relatively small sample size, from single center and geographical region, lack of control group, reliance on self-reported data, and lack of long-term follow-up. Future studies should aim to address these constraints to assess the intervention’s effectiveness. Despite these limitations, the research provides valuable insights into the potential of targeted educational interventions in promoting antimicrobial stewardship among caregivers.

## 3. Materials and Methods

### 3.1. Research Hypotheses

To fulfil the aim of the study, the following hypotheses were formulated:

1. Family caregivers who receive the antimicrobial stewardship educational intervention would have a higher level of knowledge in the post-test than pre-test.
2. Family caregivers who receive antimicrobial stewardship educational intervention would have higher level of practice in the post-test than the pre-test.

### 3.2. Research Design

Quasi-experimental, one-group pre-test and post-test design was utilized in this study to assess the effect of an educational program for family caregivers about antibiotic misuse.

#### 3.2.1. Setting

The study was carried out at one family medicine center affiliated with primary health care in El-Menoufia Governorate, the Ministry of Health and Population, Egypt. The center included three family medicine clinics (pediatric, adult, and antenatal care), a family planning clinic, an immunization clinic, two dental clinics, an investigation laboratory providing blood investigations, urine and stool analysis, a labor room, an x-ray room, a pharmacy, and an emergency room.

#### 3.2.2. Sample

A purposive sample of 300 patients was included from a population who had met the inclusion criteria of this study as follows: (1) age ≥ 18 years; (2) serving as family caregiver.

#### 3.2.3. Sample Size Calculation

The sample size acceptable for this study, using the following equation, is 300 cases. This equation was used to calculate the sample size with a significance level of 95%.

$$n=\frac{z^{2}\times p\times (1-p)/e^{2}}{1+\frac{z^{2}\times p\times (1-p)}{e^{2}\times N}}$$

where *z* = 1.96 is the *z*-score associated with the significance level chosen, *p* = 0.5 is the proportion of bullying in the population, *e* = 0.0566 is the margin of error, and *N* = 300 is the population size.

### 3.3. Tools of Data Collection

After reviewing the related national and international literature, tools for data collection were developed by the researchers to assess the antibiotic misuse among family caregivers.

Data were collected through the following tools:

1. The socio-demographic questionnaire: it included six questions on age, sex, education, marital status, family income, and occupation.
2. A structured knowledge questionnaire designed to assess family caregivers’ understanding of antimicrobial resistance and misuse. Comprising 24 questions, this instrument carefully evaluated participants’ knowledge regarding AMR, health risk, antibiotic usage, and infection prevention. Respondents indicated their answers by selecting “Yes”, “No”, or “I don’t know” for each item. The scoring system awarded 1 point for correct answers and 0 points for incorrect or “I don’t know” responses. Total knowledge scores, with a maximum of 24 points, were calculated by summing correct answers. These scores were then categorized into three tiers: low knowledge (0–7 points), moderate knowledge (8–15 points), and high knowledge (16–24 points). This nuanced assessment method provided valuable insights into caregivers’ strengths and potential knowledge gaps regarding antimicrobial resistance and proper antibiotic use.
3. Family caregiver self-reported practice regarding antimicrobial resistance and misuse. It contained 21 questions regarding the following: use of antibiotics, including previous antibiotic exposure, antibiotic use during the past year, reasons for taking antibiotics, ways of obtaining antibiotics, and reason for discontinuation of antibiotic therapy. All questions were multiple-choice, and the family caregiver could select one response.

### 3.4. Validity and Reliability

The study tools’ content validity was assessed by a panel of experts, including two community health nursing professors and one pharmacist. The alpha coefficient was used to measure agreement among expert ratings. Minor modifications were made based on panel feedback. Cronbach’s Alpha [36] was used to assess internal consistency. The knowledge questionnaire had a reliability coefficient of 0.774, while the self-reported practice questionnaire had a coefficient of 0.749.

### 3.5. Ethical Considerations

The study received approval from the Ethical Committee of the Faculty of Medicine, Menoufia University, Egypt (IRB approval number (1-2023 INT,13-2)). Participants were informed about the study’s purpose, procedures, risks, and benefits. Written informed consent was obtained, and participants’ privacy and confidentiality were protected through data anonymization and secure storage. Data collection and analysis procedures were conducted in accordance with institutional and national research committee ethical standards.

### 3.6. Procedure

The data collection process for this quasi-experimental study was meticulously executed over a six-month period, comprising three distinct phases: baseline assessment, intervention, and evaluation. In the baseline phase, participants (*n* = 300) completed a socio-demographic questionnaire and two validated instruments: a structured interview knowledge questionnaire and a self-reported practice questionnaire, both focusing on antimicrobial misuse (primary outcomes). We pilot tested these instruments for clarity and comprehension, finding an average completion time of 12.5 min (SD = 2.3). Trained research assistants collected data, adhering to standardized protocols to ensure consistency and minimize bias.

The intervention phase consisted of a rigorously designed antimicrobial stewardship educational program, developed based on the baseline assessment results and a comprehensive literature review. The program was implemented over the course of 6 weeks, with 120-minute sessions scheduled every 2 weeks. Each session employed a multimodal approach, combining interactive workshops (60 min), video-based education (30 min), and hands-on activities (30 min). Content was tailored specifically for family caregivers in primary care settings, covering both knowledge components (e.g., infectious agents, rational antibiotic use) and practice components (e.g., proper hygiene, appropriate antibiotic preparation, and storage). The same two trained facilitators conducted all sessions using a standardized curriculum to ensure fidelity.

The evaluation phase employed a post-test design, utilizing the same validated instruments from the baseline assessment to measure changes in knowledge and self-reported practices. Post-intervention data were collected within one week of the final session to minimize attrition and recall bias. Effect sizes were calculated using Cohen’s d to quantify the magnitude of the intervention’s impact.

### 3.7. Antimicrobial Stewardship Educational Program Components

The content was tailored to the specific needs and context of family caregivers in primary care settings. Antimicrobial stewardship (AMS) educational intervention encompasses both knowledge-focused and practice-focused components. The knowledge-based elements covered fundamental concepts such as infectious agents, infection spread, prevention methods, rational antibiotic use, distinctions between bacterial and viral infections, and the consequences of antibiotic resistance. Practice-oriented components addressed proper hygiene practices, appropriate antibiotic use and storage, the importance of adherence to prescribed regimens, the risks of self-medication, and effective communication with healthcare providers regarding antibiotic use.

### 3.8. Statistical Analysis

The study utilized SPSS version 26 for comprehensive statistical analysis. Descriptive statistics summarized participant demographics, knowledge, and self-reported practices. Inferential analysis employed Student’s *t*-test to assess the intervention’s effectiveness by comparing pre- and post-intervention data. Proportions of categorical variables in two or more independent groups were compared using Fisher’s exact test or Chi-square test as appropriate. *p*-values ≤ 0.05 were considered statistically significant at 95% confidence interval.

## 4. Conclusions

Educational interventions have shown significant improvements in primary healthcare settings for caregivers, promoting antimicrobial stewardship. Key improvements include increased awareness of appropriate antibiotic use and AMR, reducing self-prescription, reduced reliance on phone prescriptions, and better adherence to full antibiotic courses. However, there is still room for improvement in antibiotic sharing practices. These findings emphasize the critical need for ongoing evaluation and reinforcement of caregivers’ knowledge, practices, and capabilities in antimicrobial stewardship. The marked positive changes observed suggest that targeted educational programs could be integrated into the healthcare system, such as including them into routine physician training, developing comprehensive patient education tools, and encouraging collaboration with community health organizations. A future multicenter study with a large sample size, and a longer follow-up period is required to determine the intervention’s effectiveness.

Recommendations:

1. Conduct rigorous multicenter evaluations of educational interventions with large sample size, focusing on long-term outcomes such as sustained changes in prescribing patterns, antimicrobial resistance rates, and patient outcomes.
2. Incorporate additional methods in future research, such as direct observations or follow-up interviews, evaluating specific areas, such as self-medication and treatment adherence, to better capture whether the reported practices are sustained in real-life settings.
3. Examine a variety of tactics, including augmenting educational content with relatable examples, employing visual aids to illustrate complicated topics, and including experiential learning activities that allow caregivers to interact with the information in a more hands-on way.
4. Expand patient and public education efforts, particularly in community settings, to address misconceptions about antibiotic use and promote shared decision-making between healthcare providers and patients.
5. Explore innovative approaches to education, such as social media campaigns, and peer-to-peer learning networks, to enhance engagement and dissemination of stewardship principles.
6. Develop standardized, evidence-based educational modules and resources that can be easily adapted and implemented across different healthcare systems and geographical regions.

## Figures and Tables

**Table 1:** Distribution of family caregivers regarding their socio-demographic characteristics (*n* = 300).

Item	No.	%
Age		
18–<25	50	16.67
25–<35	78	26
35–<45	83	27.66
45+	89	29.67
Sex		
Female	294	98.0
Male	6	2.0
Education		
Can’t read and write	12	4.0
Basic education	37	12.3
Middle education	162	44.0
Higher education	89	29.7
Marital status		
Single	20	6.7
Married	240	80.0
Widow	24	8.0
Divorced	16	5.3
Occupation		
Housewife	119	39.7
Employees	141	47.0
Student	25	8.3
Other	15	5.0
Family Income		
Not enough	123	41.0
Enough	132	44.0
Enough and savings	45	15.0

**Table 2:** Distribution of the family care giver related to their knowledge in the pre and post-test (*n* = 300).

Items	Correct Knowledge				Chi-Square	*p*-Value
	Pre-Test		Post-Test			
	No.	%	No.	%		
Antibiotics’ effectiveness against viruses	129	43.0	292	97.3	7.62	0.01 *
Impact on cold/cough recovery	138	46.0	274	91.3	44.89	0.0001 *
Definition of antibiotic resistance	203	67.7	293	97.7	16.33	0.0001 *
Future mortality compared to cancer/diabetes	154	51.3	284	94.7	38.58	0.0001 *
Increasing infection resistance	196	65.3	294	98.0	19.60	0.0001 *
Self-prescribing previous antibiotics	146	48.7	279	93.0	41.62	0.0001 *
Who is affected by resistance	139	46.3	272	90.7	43.04	0.0001 *
Sharing antibiotics	152	50.7	274	91.3	34.94	0.0001 *
Effect on child immunity	263	87.7	292	97.3	1.52	0.22
Personal impact of resistance	179	59.7	286	95.3	24.62	0.0001 *
Treatment duration	181	60.3	283	94.3	22.42	0.0001 *
Antibiotic use for fever in children	225	75.0	296	98.7	9.68	0.0001 *

* Significant at *p*-value < 0.05.

**Table 3:** Distribution of the family care giver related to their practices in the pre and post-test (*n* = 300).

Items	Correct Answer				Chi-Square	*p*-Value
	Pre		Post			
	No.	%	No.	%		
What did you consider in choosing the last antibiotic?					83.9	0.0001 *
The type of antibiotic	106	35.3	53	17.7		
The manufacturing company	27	9.0	7	2.3		
Indications for antibiotic use	25	8.3	20	6.7		
The price of an antibiotic	108	36.0	209	69.7		
The side effect	26	8.7	2	0.7		
Did you change the type or dose of the antibiotic “intentionally”?					16.9	0.002 *
Yes	110	36.7	77	25.7		
No	172	57.3	217	72.3		
How did you calculate the antibiotic dose?					51.4	0.0001 *
By reading the pamphlet	30	10.0	24	8.0		
Consulted a doctor	120	40.0	197	65.7		
Consulted your pharmacist	72	24.0	44	14.7		
With family advice	37	12.3	16	5.3		
From the Internet	3	1.0	3	1.0		
From my previous experience	28	9.3	14	4.7		
Guess the dose by myself	8	2.7	0	0.0		
How often do you give your child antibiotics (without a prescription)?					36.4	0.008 *
Rarely	108	36.0	93	31.0		
Usually	99	33.0	50	16.7		
Not at all	90	30.0	155	51.7		
How often does your pediatrician prescribe antibiotics by phone?					14.1	0.001 *
Rarely	77	25.7	78	26.0		
Usually,	96	32.0	58	19.3		
Not at all	125	41.7	162	54.0		
Do you share your antibiotics with your child for similar symptoms?					1.6	0.8
Yes	67	22.3	58	19.3		
No	219	73.0	229	76.3		
When did you stop your child’s antibiotic?					13.7	0.008 *
When the disease disappears	79	26.3	55	18.3		
After the child has recovered	68	22.7	54	18.0		
After the end of the antibiotic package	18	6.0	16	5.3		
After completing the course of treatment	101	33.7	144	48.0		

* Significant at *p*-value < 0.05.

**Table 4:** Mean scores of total knowledge and practice scores of family caregiver in pre and post test (*n* = 300).

Scores	Pre-Test		Post-Test		t	*p*-Value
	Mean	SD	Mean	SD		
Total Knowledge	18.36	4.00	23.28	1.62	19.5	0.0001 *
Can’t read and write	6.25	1.35	10.21	3.14	7.2	0.0023
Basic education	8.25	1.87	12.25	2.57	10.23	0.024
Middle education	6.39	1.25	9.36	2.14	6.25	0.0052
Higher education	7.025	2.14	9.23	1.87	8.36	0.032
Total Practice	9.83	4.77	12.37	4.90	6.4	0.0001 *
Can’t read and write	2.25	0.251	5.23	1.69	5.84	0.0001
Basic education	3.84	0.924	5.84	1.32	6.24	0.0047
Middle education	4.21	0.234	6.23	1.84	8.21	0.041
Higher education	6.23	1.21	8.24	1.94	9.25	0.025

* Significant at *p*-value < 0.05.

## Data Availability

Data are available upon request.
